# Antisense gapmers selectively suppress individual oncogenic p73 splice isoforms and inhibit tumor growth *in vivo*

**DOI:** 10.1186/1476-4598-8-61

**Published:** 2009-08-11

**Authors:** Stephan Emmrich, Weiwei Wang, Katja John, Wenzhong Li, Brigitte M Pützer

**Affiliations:** 1 Department of Vectorology and Experimental Gene Therapy, Biomedical Research Center, University of Rostock, D-18057 Rostock, Germany; 2 Department of Cardiac Surgery, University of Rostock, D-18057 Rostock, Germany

## Abstract

**Background:**

Differential mRNA splicing and alternative promoter usage of the *TP73 *gene results in the expression of multiple NH2-truncated isoforms that act as oncogenes. Abundant levels of these p73 variants in a variety of human cancers correlated with adverse clinical prognosis and response failure to conventional therapies, underscoring their relevance as marker for disease severity and target for cancer intervention. With respect to an equally important role for amino-truncated p73 splice forms (ΔTAp73) and ΔNp73 (summarized as DNp73) in the tumorigenic process, we designed locked nucleic acid (LNA) antisense oligonucleotide (ASO) gapmers against individual species that were complementary to ΔEx2 and ΔEx2/3 splice junctions and a region in exon 3B unique for ΔN' and ΔN.

**Results:**

Treatment of cancer cells with these ASOs resulted in a strong and specific reduction of tumorigenic p73 transcripts and proteins, importantly, without abolishing the wild-type p73 tumor suppressor form as observed with p73-shRNA. The specific antisense oligonucleotides rescued cells from apoptosis inhibition due to overexpression of their corresponding amino-truncated p73 isoform and decreased tumor cell proliferation. Furthermore, ASO-116 against ΔEx2/3 coupled to magnetic nanobead polyethyleneimine (MNB/PEI) carriers significantly inhibited malignant melanoma growth, which correlated with a shift in the balance between endogenous TAp73 and ΔEx2/3 towards apoptotic full-length p73.

**Conclusion:**

Our study demonstrates the successful development of LNA-ASOs that selectively differentiate between the closely related p73 oncoproteins, and provide new tools to further delineate their biological properties in different human malignancies and for therapeutic cancer targeting.

## Background

The human p73 is a member of the p53 tumor suppressor family, based on substantial structural and functional homologies. Unlike p53, p73 is rarely mutated in human cancers [1]. As an early-recognized feature, the *TP73 *gene produces multiple transcripts with opposing functions. A detailed analysis of p73 in tumor cells revealed the presence of multiple N-terminally truncated isoforms (ΔN, ΔN', ΔEx2, ΔEx2/3; collectively called DNp73), which lack all or most of the transactivation domain that accounts for the tumor suppressor function of the full-length TAp73 protein. While the ΔNp73 transcript is generated from a cryptic promoter in intron 3, the majority of the amino-truncated p73 variants is produced by alternative exon splicing from the E2F1 responsive promoter in the 5'UTR upstream of the non-coding exon 1. All isoforms fail to induce cell cycle arrest and apoptosis. In addition, they act as dominant-negative competitors for DNA binding and/or heteroduplex formation with p53 and wild-type TAp73, and confer drug resistance to tumor cells harbouring wild-type p53 and/or TAp73 [2,3]. We also demonstrated that the ΔEx2/3 isoform inactivates functions of the retinoblastoma (RB) tumor suppressor protein in cell cycle and differentiation control [4,5]. By inactivating both major tumor suppressor pathways in human cells they are functionally analogous to several viral oncoproteins. In this sense, overexpression of ΔEx2/3 and ΔN has been shown to transform fibroblasts to tumorigenicity in nude mice [6,7], and drive carcinogenesis in transgenic mice *in vivo *[8].

NH2-truncated p73 forms were found frequently elevated in various types of tumor cells and primary malignancies from patients, but not in the surrounding normal tissues [9]. Beyond, increased DNp73 expression levels are strongly associated with a worse recurrence-free and overall survival of patients, and poor prognosis features such as lymph node metastasis and vascular invasion [10]. Specifically, overexpression of ΔEx2/3p73 and ΔNp73 was associated with advanced pathologic tumor stages. Likewise, alterations in the relative levels of TAp73 and the apparently tumor-specific aberrant expression of individual oncogenic p73 species, that might account for a shift in the net function of p73 from proapoptotic to prosurvival, have been shown to correlate with prognosis in some cancers. All this strongly underscores that the expression pattern of NH2-truncated p73 is an important determinant of tumor development and the cellular response to treatments, making it a biological relevant target for cancer prevention and therapy.

Although there is increasing evidence for an equally important role of individual amino-terminal p73 splice variants and ΔNp73 in the tumorigenic process, we are currently unable to evaluate their oncogenic potency under physiological conditions *in vivo*. The aim of our study was to develop mono-specific inhibitors for the majority of NH2-truncated alternative-spliced p73 isoforms that allow differential knockdown of all closely related oncoproteins in growing human tumors.

## Methods

### Cell Culture, Viruses and Fluorescence Microscopy

Human H1299 lung cancer cells, human embryonic kidney HEK 293 cells, and human WI-38 lung fibroblasts were maintained in Dulbecco's Modified Eagle Medium (DMEM; Invitrogen, Karlsruhe, Germany) supplemented with 10% fetal calf serum (Biochrom, Berlin, Germany). Human SK-Mel-29 melanoma cells were cultured in DMEM containing sodium pyruvate. Medium contained 2 mM L-glutamine, 100 μg/ml penicillin, 100 U/ml streptomycin, and 1,25 μg/ml amphotericin B. The adenoviral (Ad) vectors Ad-shGFP and Ad-shp73 have been described elsewhere [11]. Adenovirus infection was carried out at a multiplicity of infection (MOI) that allows 100% transduction of target cells. FITC and GFP imaging was performed by fluorescence microscopy at indicated time points.

### Antisense Oligonucleotides, shRNA and Transient Transfections

Oligonucleotide (ON) LNA modifications and FITC-labeling was conducted by BioTeZ GmbH. ASO sequences were the following: ASO-115 5'-**CTGT**CTGGTTCCCTGC**AGCC**-3', ASO-116 5'-**GATT**GAACTGGGCCTG**CAGC**-3', and ASO-185/451 5'-**GGAA**CTGGTGTCCCGT**GGGA**-3' (LNA bases are bold). The non-sense control ON (nsc) with 5'-**TAAC**CGTTTCTTCCTC**GTCC**-3' serving as a negative control was verified by NCBI *blastn *search. For *in vivo *experiments a scrambled ON (sc) to ASO-116 with the same base content was generated using the siRNA Wizard™ platform at  (ASO-sc: 5'-**GCCC**AGAAGGTGCCTA**GGTT**-3'). ASO transfections were performed with Lipofectamine 2000 (Invitrogen, Karlsruhe, Germany). Cotransfection experiments were carried out with 2 μg plasmid mixed with respective amounts of antisense molecules. Plasmid encoding shRNA was transfected using Effectene (Qiagen, Hilden, Germany). The RNA interference target sequence was 5'-GGCCATGCCTGTTTACAAG-3'.

### Quantitative Real-Time PCR (qPCR)

Total RNA was extracted with the NucleoSpin Kit (Macherey-Nagel, Düren, Germany) and reverse transcribed with Omniscript RT (Qiagen, Hilden, Germany) using Oligo(dT)_18 _primer. The cDNA sample was mixed with iQ SYBR Green Supermix (Biorad, München, Germany). QRT-PCR was performed on iQ5 Multicolor Real-Time PCR Detection System (Biorad, München, Germany) using 1/10 volume of RT reaction. Relative gene expression was calculated using iQ5 Optical System Software. Primer sequences are available upon request.

### Western Blotting

Cells were lysed in RIPA buffer (50 mM Tris-HCl (pH 7.2), 150 mM NaCl, 1% sodium deoxycholate, 0.1% SDS, 0.1% Triton X-100, 5 mM EDTA) supplemented with protease inhibitor mixture Complete Mini (Roche Applied Science, Mannheim, Germany) and total protein concentration was quantified by a modified Bradford assay. Equal amounts of protein were separated by SDS-PAGE, transferred to nitrocellulose membranes, and probed with anti-p73 ER-15 (BD Biosciences, Heidelberg, Germany), anti-ΔNp73 (Imgenex, San Diego, CA, USA) or anti-β-Actin (Sigma-Aldrich, Taufkirchen, Germany). Primary antibodies were detected with appropriate secondary antibody-horseradish peroxidase conjugates according to the enhanced chemoluminescence protocol.

### Hoechst 33342 Staining and Caspase-3 Activity

After treatment, cells were incubated with Hoechst 33342 at 1 μg/ml for 15 min and subjected to fluorescence microscopy. Hoechst-stained cells were harvested, counterstained with 10% trypan blue solution, and counted in a hämcytometer. Caspase-3 activity was assayed using the ApoAlert kit (Takara Bio, Saint-Germain-en-Laye, France) as described by the manufacturer. Absorbance was measured at 405 nm in a spectrophotometer.

### Cell Proliferation Assay

Cells were seeded at a density of 5 × 10^4^, serum-starved for 24 h, and transfected with PEI-ASO complexes at day 1 and 3 after plating. DNA synthesis was measured by Cell Proliferation BrdU (5-bromo-2'-deoxyuridine)-ELISA (Roche Applied Science, Mannheim, Germany) according to the instructions.

### Magnetic Nanobead (MNB)/Polyethyleneimine (PEI)/DNA Complex Formation

Branched PEI (average MW 25 000) (Sigma-Aldrich, Taufkirchen, Germany) was purified through dialysis in three changes of a 100-fold volume excess of water, lyophilized and re-hydrated before use. PEI/ASO complexes were prepared at the N/P ratio of 8. The PEI solution and DNA solution at a given N/P ratio were diluted in 5% glucose to ensure iso-osmolarity for transfection experiments and *in vivo *application. PEI was added to DNA and immediately mixed and incubated for 30 min at RT. For intratumoral injections, PEI/ASO complexes were conjugated with streptavidin-coated magnetic nanobeads (MNBs) (Promega, Mannheim, Germany) by vortexing for 30 s followed by incubation at RT for 30 min. The resulting MNB/polymer/ASO complexes are stable in aqueous solution and can be stored at 4°C for several days.

### Animal Experiments and In Vivo Imaging

Tumors were established by subcutaneous (s.c.) injection of 10^7 ^SK-Mel-29 cells into the rear flanks of 6- to 8-week-old female athymic nu/nu mice. Animals with palpable tumors were randomized in three groups to ensure uniform distribution: Group A untreated control (n = 9 tumors), group B treated with PEI/ASO-sc (n = 8 tumors), and group C treated with PEI/ASO-116 (n = 13 tumors). For magnetic force transduction, animals with tumors of diameter >8 mm were randomized into two groups (group D: MNB/PEI/ASO-sc, n = 6 tumors; group E: MNB/PEI/ASO-116, n = 10). Mice were anaesthetized by intraperitoneal injection of body-weight adapted doses of 10% ketamine and 2% xylazin. Oligonucleotide complexes were intratumorally administered at 1.5 mg/kg/day. In magnetic bead guided experiments, a magnet sized 4 × 1 mm with 1.41–1.45 T was placed closely adjacent to the tumor. Tumor volumes were measured with calipers and calculated from the longest diameter and average width by assuming a prolate spheroid shape (tumor volume = π/6 × (large diameter × [shortdiameter]^2^). The relative tumor volume (RTV) was determined using *RTV *= *V*_*i*_/*V*_0_, where *V*_*i *_is the daily-measured tumor volume and *V*_0 _is the initial tumor volume. After treatment, mice were sacrificed and representative tumor specimen of each experimental group were dissected. Total RNA was extracted and quantities of ΔEx2/3p73 and TAp73 transcripts were determined by qPCR. All animal procedures were conducted in adherenceto ethical standards and with approval of the local Animal CareCommittee.

For fluorescence detection, PEI was labeled by Oregon green 488 Protein labeling kit (Invitrogen, Karlsruhe, Germany) and injected intratumorally into mice. *In vivo *imaging was performed at 1, 8 and 24 h after injection. Images were acquired using NightOwl LB981 imaging system (Berthold Technologies, Bad Wildbad, Germany) with an exposure time of 100 s. For colocalization of the fluorescent image on the animal body, gray scale and pseudocolor images were merged. Quantification of signal intensity in all animals was performed by WinLight32 Software.

### Computational Analysis and Secondary Stucture Predictions

Human genomic sequences for the 5'UTRs of *TP73 *transcripts were obtained from ENSEMBL database. The binding energies as well as the propability matrices for single-stranded target sequences were calculated using the Sfold server application module Soligo. Each N-terminal p73 transcript was calculated with the C-terminal configurations α, β, γ and δ, and their propability diagrams were merged at the indicated nucleotide positions. *In silico *secondary structure prediction was carried out using the CLC RNA Workbench Software (CLC Bio, Aarhus N, Denmark). For each N-terminal p73 transcript C-terminal configurations α, β, γ, δ and ε were used to calculate the mRNA structure. The respective ASO epitopes at the N-termini of these mRNAs were then compared for structural identity in the ASO target sequence.

### Statistical Analysis

Statistical analysis of tumor growth in nude mice was performed with one-way analysis of variance (ANOVA). Variances in BrdU incorporation, caspase activation and viable cell counts were compared with an unpaired Student's t-test (two-sided).

## Results and Discussion

### Design of LNA-DNA gapmers against oncogenic NH2-truncated p73 isoforms

**Figure 1:**
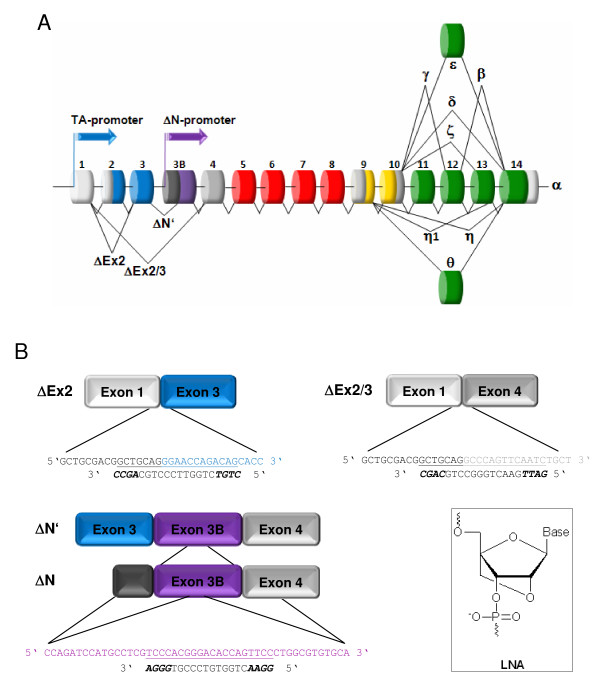
**Site-directed targeting of NH2-truncated p73 mRNAs by LNA gapmers**. (A) Structure of the human *TP73 *gene. Exons are shown as boxes and structured according to the domain: transactivation domain (blue); exon 3B-derived coding sequence (purple); DNA-binding domain (red); oligomerization domain (yellow); COOH terminus (green). C-terminal splice variations are indicated. The transcriptional start sites of the two promoter regions (TA-promoter, ΔN-promoter) are marked by arrows. Aberrantly spliced transcripts regulated by the TA-promoter are labeled ΔEx2, ΔEx2/3, and ΔN'. The unique 5' untranslated region of the ΔN transcript is colored grey. (B) Binding sites of gapmer ASOs in different DNp73 transcripts. ASO-115/ΔEx2p73, ASO-116/ΔEx2/3, and ASO-185/451 directed against ΔN'p73 and ΔNp73 mRNA. LNA bases are in bold.

Differential mRNA splicing and alternative promoter usage of the human *TP73 *gene results in the expression of multiple N-terminally truncated forms (ΔEx2, ΔEx2/3, ΔN', ΔN; Figure 1A) that act as oncogenes. Current evidence suggests an equally important role for individual p73 splice variants and ΔNp73 in the tumorigenic process, but there is no attempt so far for their selective silencing without concomitant reduction of the apoptotic TAp73 form. In order to develop mono-specific inhibitors for each amino-terminal isoform, we used a modified antisense technology. According to previous reports indicating that ONs with locked nucleic acids (LNA) at their 3'- and 5'-ends efficiently recruit RNase H and promote degradation of target RNA [12,13], we designed 20-mer LNA-DNA gapmers with a central DNA region of 12 nucleotides and 3'-5' ends of four LNA monomers. LNA bases have been shown to confer resistance to nucleases when incorporated at the 5' and 3' ends of oligomers [14]. Antisense specificity for ΔEx2p73 and ΔEx2/3p73 was achieved by using the splice junction of each exon deletion variant (Figure 1B). The ASO targeting ΔN' and ΔN was directed against a region in the intron-derived exon 3B, which is unique to both isoforms.

The optimal target sequence was determined *in silico *by three parameters: (a) a probability profile for single-stranded regions in the mRNA, which serve as docking stations for initial oligo annealing [15,16], (b) a minimum free energy model of the secondary structure of the mRNA with a more recent compilation of Turner's free energy parameters [17], and (c) the binding energies for a given ASO sequence on the mRNA [18]. The probability plot for ΔEx2p73 mRNAs indicates single-strands downstream of nucleotide (nt) position 120 and around position 130 [see Additional file 1A]. This was confirmed by secondary structure prediction for ΔEx2p73 transcripts, where two bulges of more than 4 bases were present at positions 121 and 130, exposing the splice junction for oligo binding [see Additional file 1E]. Since mRNA 3D-structure depends predominantly on nearest neighbour interactions, alterations in the C-terminus may affect folding of N-terminal domains [19]. Therefore, structure predictions were carried out for several C-terminal isoform configurations. Binding energies for ASO-115 (number indicates starting base of ASO position on the mRNA) targeting ΔEx2p73 mRNA reached values around -8 kcal/mol [see Additional file 2]. According to the Soligo algorithm using ≤ -8 kcal/mol as default filter criteria for potent ASOs, the values for ASO-115 were considered sufficient. Similar results were obtained for ASO-116. Analysis for single-stranded sections revealed a high probability for unpaired mRNA at the splice junction of ΔEx2/3p73 transcripts, which was confirmed by the predicted structure model [see Additional file 1B, F]. In the ΔNp73 and ΔN'p73 ASO, where the target region is of different location in each transcript owing to distinct transcriptional and mRNA maturation processes, ASO-185/451 denotes the starting positions for the ON in both isoforms. The probability plots and secondary structures of ΔNp73 showed the minimum of 4 unpaired bases, which are sufficient for initial oligo annealing [see Additional file 1C, G] [19]. Likewise the predicted epitopes and probability plots for ΔN'p73 transcripts suggest mRNA accessibility at the target region of ASO-185/451 [see Additional file 1D, H].

### ASOs induce selective suppression of target RNAs

**Figure 2:**
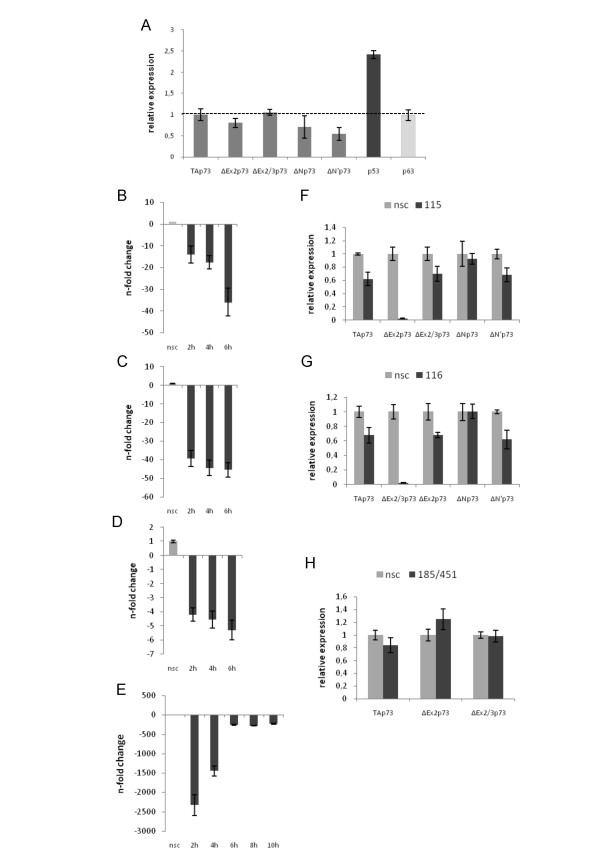
**Efficacy and specificity of ASOs for endogenously upregulated p73 transcripts**. (A) Quantitative RT-PCR of endogenous expression of TAp73 and amino-truncated p73 transcripts relative to p53 and p63 in HEK293 cells. The broken line indicates the baseline level of DNp73 transcripts. Cells were transfected with 250 nM of ASO-115 (B, F), ASO-116 (C, G), and ASO-185/451 (D, E, and H). Antisense effects on transcript levels were quantitated by real-time PCR at indicated time points after treatment. Fold expression was calculated after normalization with RPS9 relative to non-specific control-ASO (nsc). Averages and standard deviations of at least three independent experiments are shown.

To ensure efficient internalization of the ASOs into target cells, we determined the amount of oligonucleotide uptake after 8, 16 and 24 h of transfection using a concentration range between 100–250 nM fluorescein isothiocyanate (FITC) labeled ASO. All LNA-DNA gapmers showed a similar high transfection efficiency in HEK293, H1299, and SK-Mel-29 cells overexpressing individual DNp73 isoforms, and similar intracellular distribution patterns with clear nuclear, perinuclear, and diffuse cytosolic staining (data not shown). The capacity and specificity of the designed antisense gapmers to reduce endogenous levels of ΔEx2, ΔEx2/3, ΔN', and ΔN transcripts was subsequently analyzed by qRT-PCR in HEK293 containing all N-terminally truncated isoforms at higher levels (Figure 2A). Based on previous results indicating that RNase H enzymatic activity is stimulated as early as 1 h after ASO treatment [20], antisense molecules were transfected at a concentration of 250 nM into HEK293 cells and gene silencing efficiencies have been monitored over 2, 4, and 6 h after treatment. The effect of a non-sense control ON is shown for comparison. In these cells, ASO-115, 116, and 185/451 directed against the splice forms ΔEx2, ΔEx2/3 or ΔN' induced the strongest antisense effect after 6 h compared to the nsc-gapmer. At this time point, we measured a 35- to 45-fold reduction of gene expression for both ASO-115 (Figure 2B) and ASO-116 (Figure 2C), whereas ASO-185/451 promoted a weaker (5-fold) but still significant suppression of the ΔN' mRNA (Figure 2D). Quantitative analysis of the silencing efficiency of the ASO-185/451 antisense gapmer against its second target transcript, revealed a substantially higher inhibitory effect with a more than 2000-fold reduction of the ΔN mRNA level already 2 h following treatment relative to the nsc-ASO (Figure 2E). Repeated experiments provided similar results with rendering the ΔNp73 transcript barely detectable during the first 4 h post transfection. The following time points showed a constant 225- and 279-fold suppression, suggesting a prolonged duration of gene silencing upon amplified initial repression intensity.

The main goal of our study was to design ASOs that allow specific knockdown of individual oncogenic p73 forms. As demonstrated in Figure 2F, ASO-115 induced a complete decrease of ΔEx2p73 mRNA to 0.03% of the control, whereas expression of TAp73 and other non-targeted p73 variants remained largely unaffected. Similar data was obtained with the ASO-116 against ΔEx2/3p73 (Figure 2G). Interestingly, both ASOs targeting the splice junction of exon 1 have very little effect on ΔNp73 transcripts, which lack exons 1 to 3 due to alternative promoter usage. Analogous to this, ASO-185/451 revealed no silencing impact on the ΔEx2 and ΔEx2/3 transcripts because of the absence of exon 3B in these variants (Figure 2H). These results indicate that all developed ASOs exhibit a selectively high inhibitory efficiency against their target, resulting in no or low knockdown of other p73 isoforms. Comparable high isoform-specific knockdown activities of these ASOs were also observed in H1299 tumor cells with amino-truncated p73 transcripts upregulated [see Additional file 3].

### Effect of site-directed targeting on DNp73 protein level

**Figure 3:**
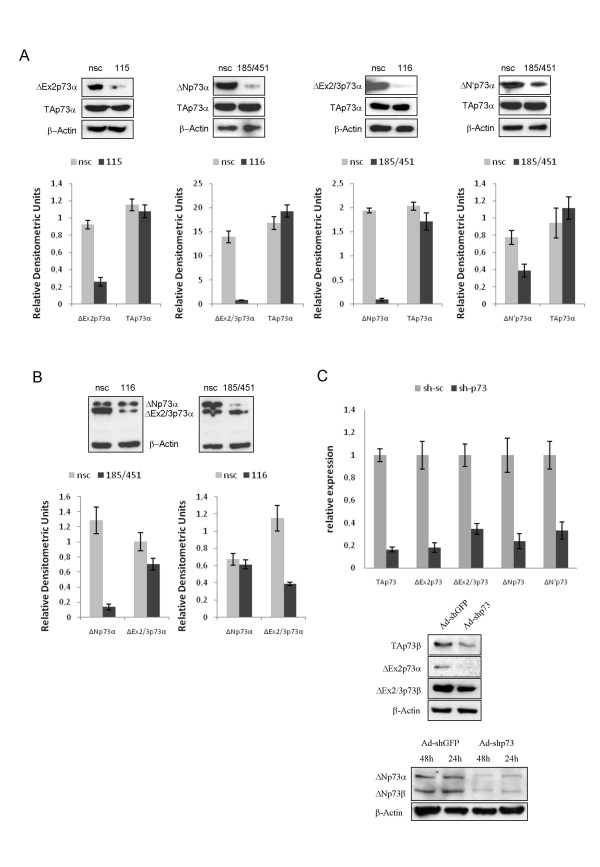
**Knockdown effect of NH2-isofom specific ASOs on protein level compared to p73-shRNA**. (A) Western blot analysis showing the levels of full-length TAp73 and amino-truncated p73 forms 24 h following cotransfection of 2 μg p73 expression plasmids and 500 nM antisense or nsc LNA-DNA gapmers. Actin was used as a loading control. Protein bands were quantitated in relative software units by the Bio-Imaging-Analyzer (Fuji) using the TINA program (shown as fold induction or reduction, respectively, normalized to actin control bands. (B) Protein levels of ΔEx2/3 and ΔNp73 in H1299 cells cotransfected with ΔEx2/3p73 and ΔNp73 expression plasmids along with ASO-116 (left) or ASO-185/451 (right) compared to control-ASO. Relative densitometric units analyzed as described in A are shown in the bottom panel. (C) QPCR indicating the endogenous expression levels of p73 isoform mRNAs normalized to RPS9 in HEK293 cells at 48 h after transfection with 1 μg p73-shRNA encoding plasmid relative to levels in control sh-sc treated cells (set as 1). Bar graphs show results from three independent experiments. Data are the mean ± SD. Immunoblot of H1299 cells with endogenous ΔN levels and transfected with 1 μg of expression plasmids for TAp73, ΔEx2, and ΔEx2/3. Cells were treated with Ad-shp73 or Ad-shGFP at moi 20. Forty-eight hours after infection, cells were lysed, and extracts were probed with anti-p73 (ER-15) antibody. β-actin was used for equal loading (bottom panel).

As endogenous levels of all amino-truncated p73 proteins are hardly detectable in a single cell line, expression plasmids containing the transcript-specific 5'UTRs for the distinct isoforms were cotransfected with 500 nM of the antisense molecules into H1299 cells and analyzed by Western blot. Due to the inherently high stability of the oncogenic proteins, we measured their level in specific LNA gapmer- and control ON-treated cells after 24 h. The levels of full-length TAp73 and DNp73 forms in H1299 cells treated with LNA-DNA are shown in histograms. Following a single transfection, ASO-115 strongly reduced the level of ΔEx2p73 protein up to 30% of control, but not TAp73 (Figure 3A). A nearly complete disruption of the ΔEx2/3p73α protein to less than 5% of the nsc-oligonucleotide was achieved by the treatment with ASO-116. ASO-185/451 but not the negative nsc-ASO significantly suppressed both targets on protein level, although protein derived from the ΔNp73α plasmid was reduced more efficiently than ΔN'. These findings correlate with the obtained qPCR results, where ASO-185/451 targeting ΔNp73 and ASO-116 directed against ΔEx2/3 showed the strongest suppression activity. In addition, we cotransfected the two different amino-terminal isoforms ΔEx2/3 and ΔNp73 along with one of the antisense molecules. As demonstrated in Figure 3B, ASO-116 and vice versa ASO-185/451 inhibited their target in a highly specific manner, while protein expression of the second ΔNp73 or ΔEx2/3p73 isoform in each case remained unchanged. Importantly, consistent with the PCR data, none of the designed LNA gapmers against the oncogenic variants of p73 has an obvious effect on the amount of TAp73 protein (Figure 3A).

This is in sharp contrast to the effect of a p73-shRNA. The use of RNAi is restricted to sequences that fullfill special criteria, such as the GC content, A at position 19 or absence of internal repeats [21]. According to these parameters, the target sequence of the p73-shRNA is located in exon 5 of the transcript. In contrast to the data observed with the NH2-truncated isoform-specific gapmers, transfection of HEK293 cells with a plasmid encoding shRNA against p73 resulted in a general silencing of TAp73 and oncogenic forms up to 80% of the scrambled-shRNA, with the strongest potency against the wildtype transcript (Figure 3C, upper panel). This unspecific inhibitory effect was also confirmed on protein level. In H1299 cells infected with an adenoviral vector expressing p73-shRNA, we found all N-terminal isoforms and TAp73 equally suppressed at 48 h after infection (Figure 3C, bottom panel) compared to the control virus Ad-shGFP. From these results, we conclude that site-directed targeting of ΔEx2 and ΔEx2/3 splice junctions and a unique region in Exon3B for ΔN' and ΔN by antisense ON is a feasible technique for selective knockdown of individual p73 oncogenes.

### Antitumorigenic activity of DNp73 ASOs

**Figure 4:**
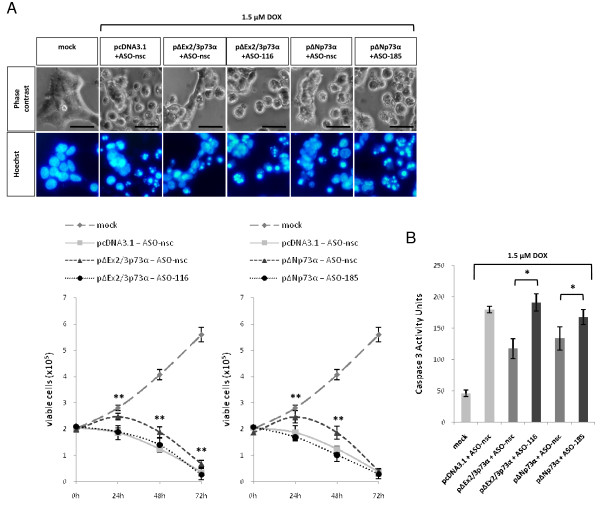
**Induction of apoptosis in WI-38 fibroblasts by ASO-specific inhibition of DNp73**. (A) Cells cultured in medium containing 1.5 μM doxorubicin (DOX) were stained with Hoechst 33342 at 48 hours after cotransfection. Apoptotic cells show the typical features of membrane blebbing, cell shrinkage, and nuclear condensation (upper panel). The amount of viable cells cotransfected with ΔEx2/3p73α or ΔNp73α in the absence and presence of specific ASO was counted at indicated time points after trypan blue exclusion. Black bars correspond to 50 μm. (B) Detection of caspase-3 activity of cells treated as in A. Bar graphs show the mean ± S.D. of three independent experiments. Asterisks denote statistical significant *p*-values: * (*p *< 0.05); ** (*p *< 0.005).

Previous studies have shown that NH2-truncated p73 species inhibit apoptosis by p53 and TAp73 in cancer cells [3,7,22]. Consequently, the proapoptotic function of these proteins could be restored by depleting the pool of antagonistic p73 proteins. After having shown that the designed ASOs specifically silence DNp73 forms, we determined whether they also influence cellular survival. Figure 4A shows that ΔEx2/3p73α and ΔNp73α overexpression markedly reduced the response of normal fibroblasts to genotoxic damage induced by doxorubicin compared to pcDNA3.1 + ASO-nsc treated cells with clear apoptotic features. In contrast, the apoptosis antagonizing effect of both oncogenic p73 forms significantly declined after cotransfection of their specific ASO resulting in increased cell killing over time. Accordingly, while the DNp73 isoforms partially rescued WI-38 cells from DNA damage, isoform-specific ASOs substantially enhanced caspase 3 activity (Figure 4B).

**Figure 5:**
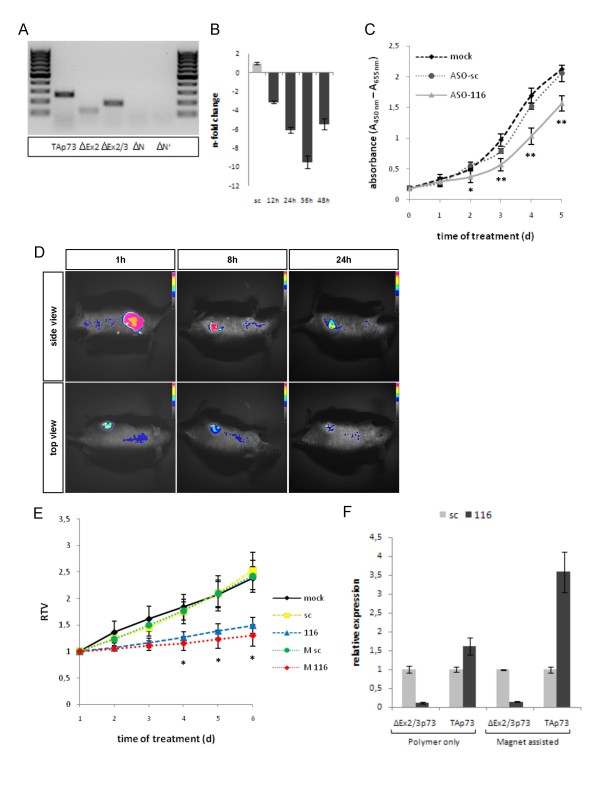
**Activity of PEI/ASO-116 in SK-Mel-29 cells and effect on tumor xenografts *in vivo***. (A) Semiquantitative RT-PCR analysis of endogenous p73 isoforms in melanoma cells. (B) Quantification of ΔEx2/3p73 transcript levels in melanoma cells transfected with 250 nM PEI/ASO-116 by real-time PCR at indicated time points. Expression was normalized to RPS9. Fold changes are relative to ASO-nsc. Data represent the mean ± S.D. of three independent experiments. (C) BrdU incorporation of cells transfected with PEI/ASO-116- or ASO-sc on day 1 and 3 was measured by ELISA. Significant differences are labeled with asterisks (* *p *< 0.01; ** *p *< 0.005). (D) *In vivo *imaging of PEI/ASO complex 1, 8, and 24 h after intratumoral injection. The fluorescence image (pseudocolor) was overlaid on the photographic image. Intensity of fluorescent signal from Oregon green 488 labeled ASO is shown in top and side view. (E) Relative tumor volumes (RTV) of SK-Mel-29 xenografts injected with PEI/ASO (sc and 116) or MNB/PEI/ASO (M sc and M 116) at a daily interval (*p *< 0.001). Significant differences between M 116 and 116 are labeled with asterisks (*p < 0.05). (F) ΔEx2/3p73 and TAp73 transcript levels in tumor tissues after 6 day treatment shown in E were quantitated by qRT-PCR. Expression was normalized to RPS9. Fold changes are relative to scrambled controls (set as 1). Bars indicate mean values ± S.D. of n = 4 for each treatment.

Finally, in an effort to corroborate the role of single amino-truncated p73 species in the tumorigenic process, we tested DNp73-specific ASOs in tumor xenografts established from SK-Mel-29 melanoma cells. These rapidly growing tumors, exhibiting major defects in p53 related apoptosis pathways [23], were selected for our studies due to their high level expression of endogenous ΔEx2/3 (Figure 5A), which has so far been implicated in the induction of tumor growth when overexpressed [7,8]. For efficient delivery of antisense molecules, ASO-116 was complexed to synthetic nanopolymer PEI [24]. Analysis of the PEI/ASO-116 polyplexes in cultured SK-Mel-29 cells showed a strong 6-to 9-fold decrease of target mRNA level after transfection (Figure 5B). This inhibitory effect on ΔEx2/3p73 expression was associated with a significantly reduced proliferation rate by PEI-ASO-116 over 5 days compared to untreated or PEI/ASO-sc treated cells as demonstrated by BrdU-ELISA (Figure 5C). Our data confirm that p73 species-specific ASOs conjugated to PEI are biologically active. Using fluorescence-labeled PEI/ASO-116, signal intensity peaked at 1 h after intratumoral injection, indicating ASO distribution within the whole tumor (Figure 5D). Although the complex decreased over time, a stabil fraction remained detectable in the tumor after 24 h, which is sufficient to ensure continuous availability of ASO over the treatment period at daily administration.

In order to improve *in vivo *administration, magnetic nanobeads (MNBs) were used to prevent diffusion of the coupled PEI/ASO complex from the injection site, and PEI/ASO-116 coated to MNBs versus PEI/ASO-116 alone were tested for their antitumoral effect in presence of a magnet implanted near the tumors. Consistent with the inhibitory effect on cell proliferation illustrated in Figure 5C, tumor growth curves revealed a robust decrease in growth rates when treated with ASO-116 independent of the mode of delivery (Figure 5E). However, with increasing treatment tumors injected with ASO-116 under magnetic force-guidance were significantly smaller than those treated with PEI/ASO-116 alone. This demonstrates that by keeping the ASO concentrated in the tumor, we could enhance its specific therapeutic efficacy. Quantification of p73 transcripts in the tumors after final injection revealed an equally strong suppression of ΔEx2/3p73 for the PEI- and MNB/PEI-delivered antisense oligonucleotide (8.5-fold and 7-fold) compared to the control groups (Figure 5F). Of note, enforced antitumorigenic activity of ASO-116 directly correlated with the upregulation of the tumor suppressive TAp73 form, which showed a more than 2 times higher increase when tumors were subjected to magnetic force-guided ASO-116 treatment. These data suggest that inhibition of TAp73 in response to increased ΔEx2/3p73 expression in growing tumors is the mechanism responsible for ΔEx2/3p73-mediated tumor growth.

Based on present studies, NH2-truncated p73 isoforms are upregulated in the majority of tumors with a concomitant rise of TAp73 [9], suggesting that the specific ratio between the apoptotic TAp73 form and different dominant-negative p73 variants determines the functional outcome of p73 and the cell fate. In fact, alterations in the relative levels of truncated p73 and TAp73 and/or p53 correlate with therapy failure and poor prognosis in many cancers [10,25,26]. In this regard, downregulation of the ΔNp73 species by antisense technique has been shown to alleviate its suppressive action and to enhance p53/TAp73-mediated apoptosis in cancer cells in response to chemotherapy [3]. In turn, mice harbouring a specific knockout of TAp73 are tumor-prone and sensitive to carcinogens. Tomasini et al. reported that 73% of TAp73^-/- ^mice spontaneously develop malignancies, thereby establishing TAp73 isoforms as bona fide tumor suppressors [27]. Furthermore, this group showed that TAp73 is regulating the spindle assembly checkpoint (SAC) complex, suggesting that SAC impairment leads to genomic instability and aneuploidity in TAp73-deficient cells. Hence, the ability to induce and maintain proper mitotic arrest could be a mechanism of TAp73-mediated anti-tumorigenicity [28]. Both studies emphasize the functional importance of an imbalanced TAp73:DNp73 ratio. Taking into account that the expression levels of individual DNp73 species might be tumor-specific and particularly alternative-spliced isoforms ΔEx2/3, ΔEx2, and ΔN' that mimic the TAp73 knockout are predominantly expressed in many cancers including malignant melanoma [10,29-33] rather than ΔNp73, specific inhibitors for these potential oncogenes are needed. In this study, we show that selective knockdown of a single p73 splice product leads to the inhibition of melanoma tumor growth, and at the same time to the induction of TAp73, underscoring that the ratio between TAp73 and ΔEx2/3p73 accounts for its oncogenic activity. Consistent with the induction of apoptosis after ΔNp73 knockdown, ASO-mediated suppression of ΔTAp73 isoforms was sufficient to abrogate apoptosis resistance to chemotherapy mediated by ectopically expressed ΔTAp73. Enhanced chemosensitivity by inhibition of ΔTAp73 spliced isoforms was recently demonstrated in neuroblastoma using the cyclooxygenase inhibitor celecoxib, which blocks both ΔEx2/3p73 and ΔEx2p73 without isoform selectivity [34].

One of the most required desirements for LNA-ASOs is their efficient delivery to target cell nuclei. Polymers of cationic polyethyleneimine are well-studied compounds that improve the *in vitro *and *in vivo *penetrance of ASOs to cells and tissues [35], yet PEI-mediated DNA transfer is not tumor directed. Thus, we aimed at constructing targeted non viral vectors based on magnetic force-guided polyplexes by coating PEI/ASO to nanoparticles that allow enforced cellular uptake by complex concentration at the tumor site. As a result, mice that received accumulating intratumoral injections of MNB/PEI-ASO complex against ΔEx2/3 showed a stronger degree of tumor growth inhibition than those treated only with PEI/ASO, indicating that increased antisense activity, substantiated by a marked induction of TAp73, correlates with higher transfection efficiency in tumor cells. Since the release of p73/p53 tumor suppressor function from ΔTAp73-mediated inhibition has been linked to chemosensitivity [36], the use of oncogenic p73 ASOs as a therapeutic strategy for cancer treatment could be further improved by combination with other genotoxic agents.

## Conclusion

We have developed LNA-modified ASOs that downregulate individual aberrantly expressed ΔEx2, ΔEx2/3, ΔN', and ΔN p73 forms in neoplastic cells and growing tumors with high specificity. ASO-mediated modulation of endogenous isoform expression resulted in tumor growth inhibition *in vivo *accompanied by the induction of apoptotic TAp73. The data of this study support the application of NH2-truncated p73 inhibitors as valuable tools to delineate their biological role in human cancers and as anticancer agents.

## Competing interests

The authors declare that they have no competing interests.

## Authors' contributions

SE and BMP designed the study and wrote the manuscript. SE, KJ and WW performed molecular studies. WL provided purified polymers. SE performed statistical analysis and data interpretation. All authors read and approved the final manuscript.

## Supplementary Material

Additional file 1 **Design of DNp73-ASOs**. Probability blots are shown as overlays of four C-terminal isoform constellations. The respective ASO target position is illustrated by the blue-shaded dashed line in each plot. ΔEx2p73 (A), ΔEx2/3p73 (B), ΔNp73 (C), and ΔN'p73 (D). (E-H) Secondary structure models indicate the ASO epitopes in the corresponding target transcripts, specific splice junctions in the epitopes of p73ΔEx2 and ΔEx2/3 are labelled by double arrows. Predicted free energies ΔG are 917,0 kcal/mol for ΔEx2p73 (E), 866,3 kcal/mol for ΔEx2/3p73 (F), 901,2 kcal/mol for ΔNp73 (G), and 1056,8 kcal/mol for ΔN'p73 (H). Click here for file

Additional file 2 ***In silico *calculated ASO binding energies**. The data provided represent the binding energies of antisense oligonucleotides for each N-terminal p73 transcript with different C-terminal configurations. Click here for file

Additional file 3 **Specific knockdown effect of ASO-115 and ASO-116 on DNp73 mRNA levels in tumor cells**. H1299 cells with endogenously upregulated amino-truncated p73 transcripts were treated with ASOs as shown in Figure 2. Fold expression was calculated after normalization with RPS9 relative to non-specific control-ASO (nsc). Bars indicate the mean ± S.D. of three independent experiments. Click here for file
