# Supplementary figures and images for: Antisense gapmers selectively suppress individual oncogenic p73 splice isoforms and inhibit tumor growth in vivo
Source: Mol Cancer. 2009 Aug 11;8:61. doi: 10.1186/1476-4598-8-61 (PMC2734544; doi:10.1186/1476-4598-8-61)

## Slide 1
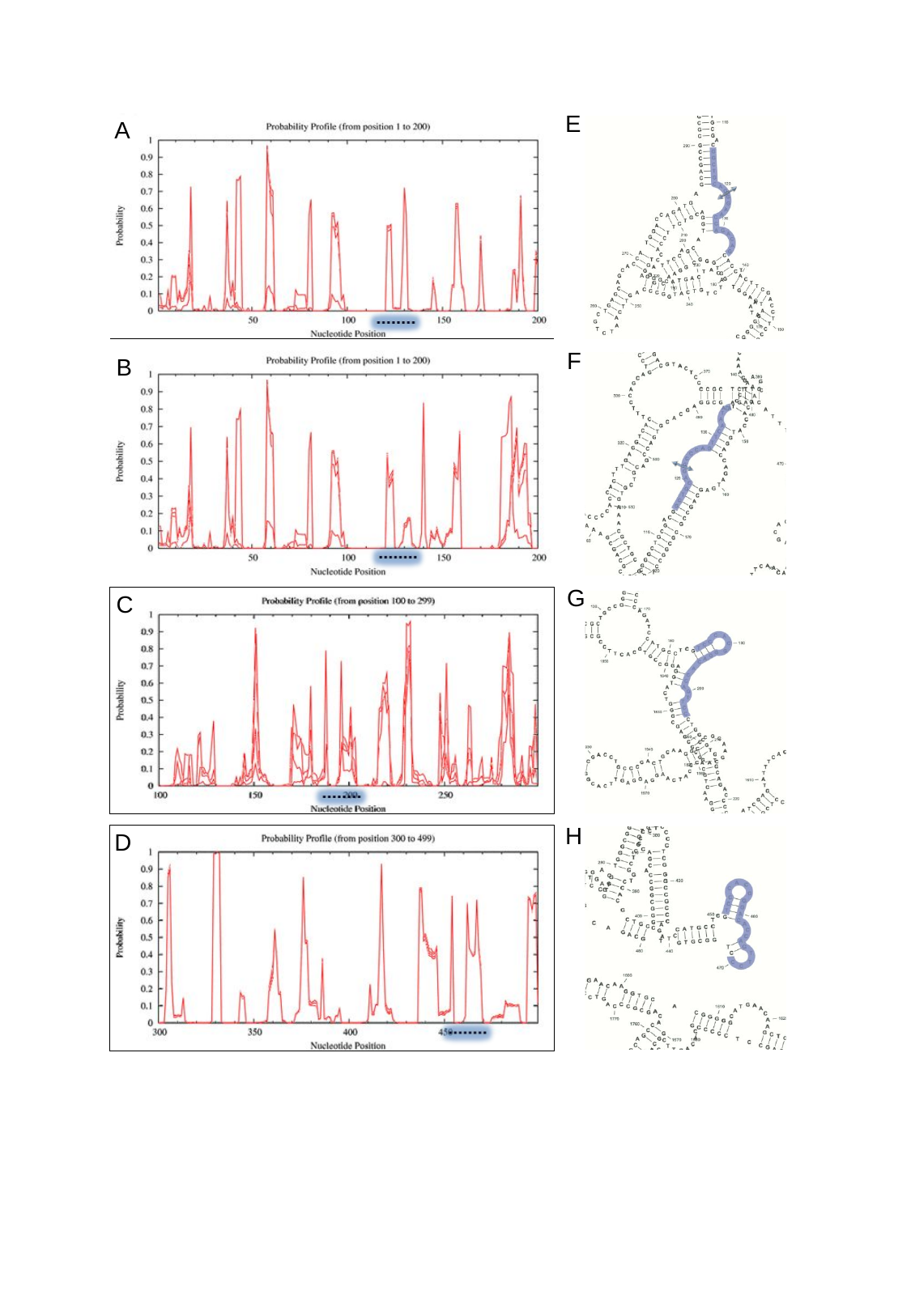

E
A
F
B
G
C
H
D

Supplement: Additional file 1 — Design of DNp73-ASOs. Probability blots are shown as overlays of four C-terminal isoform constellations. The respective ASO target position is illustrated by the blue-shaded dashed line in each plot. ΔEx2p73 (A), ΔEx2/3p73 (B), ΔNp73 (C), and ΔN'p73 (D). (E-H) Secondary structure models indicate the ASO epitopes in the corresponding target transcripts, specific splice junctions in the epitopes of p73ΔEx2 and ΔEx2/3 are labelled by double arrows. Predicted free energies ΔG are 917,0 kcal/mol for ΔEx2p73 (E), 866,3 kcal/mol for ΔEx2/3p73 (F), 901,2 kcal/mol for ΔNp73 (G), and 1056,8 kcal/mol for ΔN'p73 (H). [file 1476-4598-8-61-S1.ppt]

## Slide 1
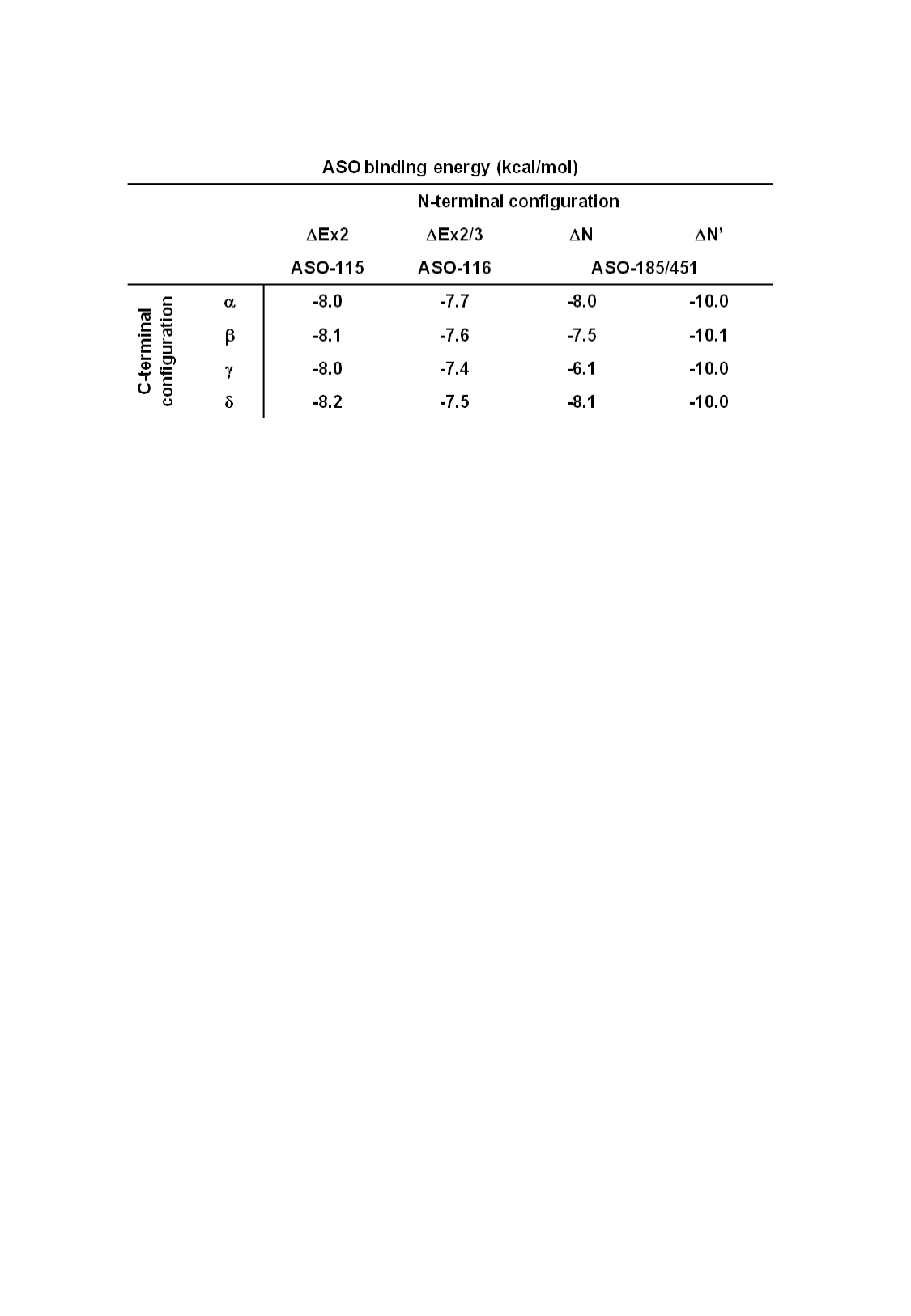

Supplement: Additional file 2 — In silico calculated ASO binding energies. The data provided represent the binding energies of antisense oligonucleotides for each N-terminal p73 transcript with different C-terminal configurations. [file 1476-4598-8-61-S2.ppt]

## Slide 1
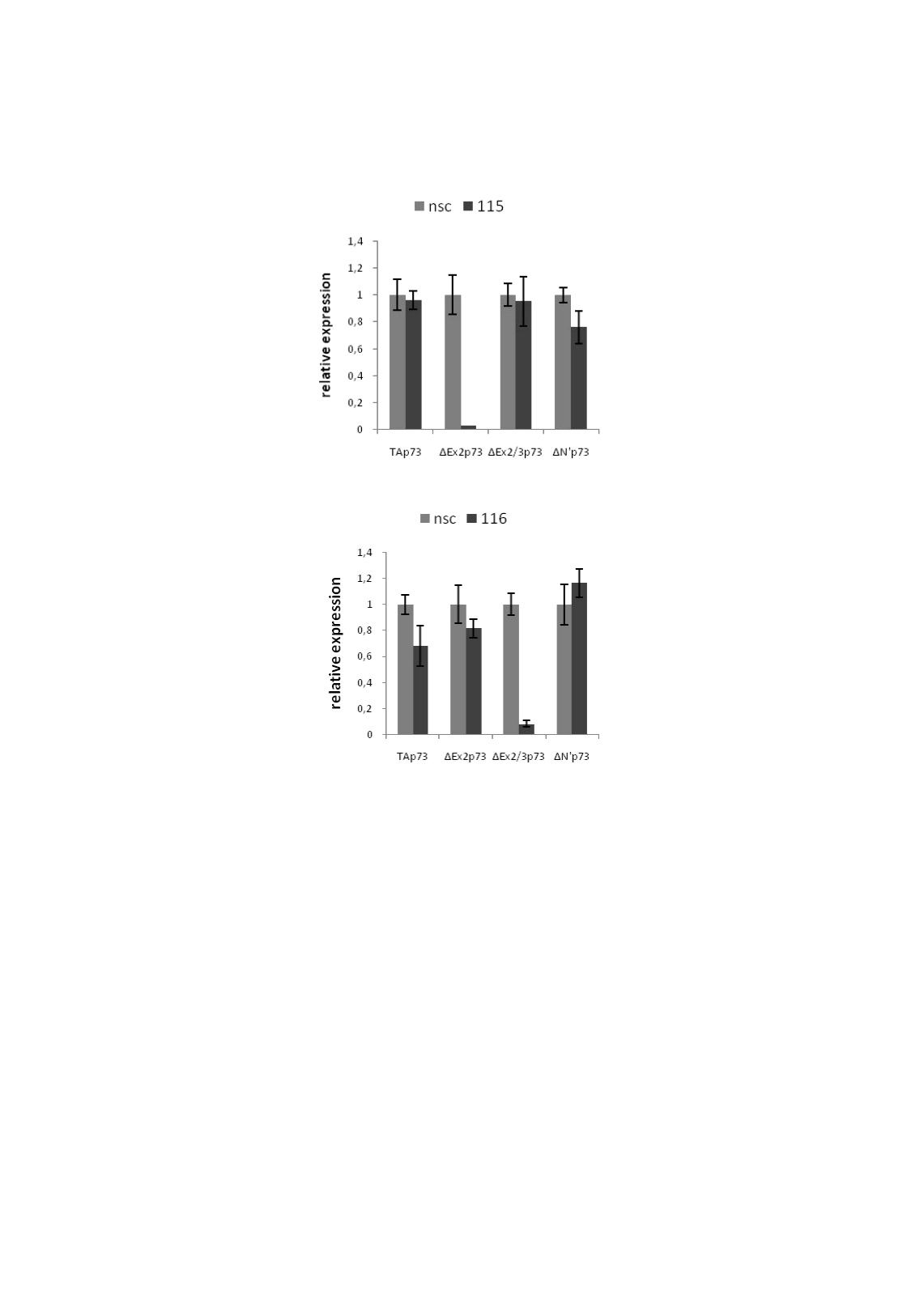

Supplement: Additional file 3 — Specific knockdown effect of ASO-115 and ASO-116 on DNp73 mRNA levels in tumor cells. H1299 cells with endogenously upregulated amino-truncated p73 transcripts were treated with ASOs as shown in Figure 2. Fold expression was calculated after normalization with RPS9 relative to non-specific control-ASO (nsc). Bars indicate the mean ± S.D. of three independent experiments. [file 1476-4598-8-61-S3.ppt]
